# A Comparison of Conventional and Ultrasound-Assisted BCR Sequential Extraction Methods for the Fractionation of Heavy Metals in Sewage Sludge of Different Characteristics

**DOI:** 10.3390/molecules27154947

**Published:** 2022-08-03

**Authors:** Malwina Tytła, Kamila Widziewicz-Rzońca, Zuzanna Bernaś

**Affiliations:** Institute of Environmental Engineering, Polish Academy of Sciences, 34 M. Skłodowskiej-Curie St., 41-819 Zabrze, Poland; kamila.rzonca@ipispan.edu.pl (K.W.-R.); zuzanna.bernas@ipispan.edu.pl (Z.B.)

**Keywords:** heavy metals, sewage sludge, conventional BCR sequential extraction (CSE), ultrasound-assisted BCR sequential extraction (USE), ultrasound, fractionation

## Abstract

The purpose of this study was to determine the heavy metal (HM: Cd, Cr, Cu, Ni, Pb, Zn, and Hg) content in particular chemical fractions (forms) of sewage sludge with different characteristics (primary and dewatered sludge) using conventional (CSE) and ultrasound-assisted (USE) BCR sequential extraction methods (Community Bureau of Reference, now the Standards, Measurements and Testing Programme). The concentrations of HMs were determined using inductively coupled plasma optical spectrometry (ICP-OES). Only mercury was assayed with cold vapor atomic absorption spectrometry (CVAAS). Ultrasound treatment was conducted in the ultrasonic bath (Sonic 5, Polsonic). The optimal sonication time (30 min) was determined using ERM-CC144 (Joint Research Center; JCR) certified reference material. The conducted experiment revealed that the use of ultrasound waves shortened the extraction time to 4 h and 30 min (Stages I to III). The recoveries (R_M_) of heavy metals ranged from 62.8% to 130.2% (CSE) and from 79.8% to 135.7% (USE) for primary sludge, and from 87.2% to 113.2% (CSE) and from 87.8% to 112.0% (USE) for dewatered sludge. The only exception was Hg in dewatered sludge. The conducted research revealed minor differences in the concentrations and fractionation patterns for Cd, Ni, and Zn extracted from sludge samples by the tested methods. However, it was confirmed that the above findings do not significantly affect the results of a potential ecological risk assessment (with minor exceptions for Cd and Zn in the primary sludge), which is extremely essential for the natural use of sludge, and especially dewatered sludge (the final sludge). The shorter extraction time and lower energy consumption prove that ultrasound-assisted extraction is a fast and simple method for HM fractionation, and that it provides an alternative to the conventional procedure. Therefore, it can be considered a “green method” for the assessment of the bioavailability and mobility of heavy metals in solid samples.

## 1. Introduction

Municipal sewage sludge is a by-product of multi-stage wastewater treatment that contains substances such as nitrogen, phosphorus, potassium, calcium, and magnesium (nutrients), as well as organic matter (OM), which are required in large amounts for the proper growth of plants [1,2,3]. Precisely for these reasons, sewage sludge is used as a soil amendment or fertilizer [4]. Unfortunately, it also contains pathogens, poorly biodegradable organic compounds, and potentially bioaccumulative, toxic, and carcinogenic elements, such as heavy metals (HMs), which pose potential safety hazards to soils, plants, animals, and most of all to humans [1,5,6,7,8]. Taking into account the pace of population growth, as well as the development of industry and new technologies, it is predicted that the amount of sewage sludge will increase rapidly, while European Union (EU) regulations are becoming more stringent. Considering the above facts, the determination of heavy metals in sewage sludge is becoming more and more important, especially because these contaminants are not biodegradable in the natural environment [4]. This means that metals can accumulate in soil, then enter the food chain through crops, and finally concentrate in the environment. However, the total content of heavy metals is not a reliable indicator of the negative effects of their impact on living organisms and the environment [2,9]. The best way to obtain more specific information about the bioavailability, occurrence, and potential mobility of these elements is to carry out chemical sequential extraction [5,10].

Over the years, many scientists have studied different extraction methods and tested various chemical reagents and conditions to find the best procedure for the determination of the chemical fractions (forms) of metals in various environmental matrices [11,12,13,14,15,16,17]. Chemical sequential extraction procedures have been applied to solid samples, such as soils, sediments, sewage sludge, and related materials [5,6,12,13,18,19]. Nowadays, the most commonly used method of sequential extraction is the three-stage procedure proposed by the Community Bureau of Reference (BCR, now the Standards, Measurements and Testing Programme), which is a modification of the Tessier method developed in 1979 (a five-stage procedure) [11]. The BCR sequential extraction allows for the determination of the constituents of sewage sludge-bound metals, and enables an assessment of their ability to migrate to the environment, which is essential in the case of the natural use of sludge [20]. However, this method is extremely time-consuming. Therefore, it is necessary to reduce the many-hours-long sample shaking procedure and/or replace it with another process that would enable results similar to those obtained with the conventional method to be achieved. The most promising and beneficial method seems to be ultrasonication (sonication), which is based on the cavitation phenomenon [5,21,22]. It has been shown that the cavitational effect generated by ultrasound can fragment particles and cause microcracks, which in turn accelerate various physicochemical processes, i.a., digestion, dissolution, extraction, or leaching [5,18,19,22]. Ultrasound treatment is most often conducted in an ultrasonic bath, where many samples can be treated simultaneously [5,18,19]. Taking into account that ultrasound treatment shortens the extraction time and reduces the energy consumption of the process, it can be considered a “green method” for the assessment of the bioavailability and mobility of heavy metals in solid samples [18,23].

In conclusion, it can be assumed that ultrasound-assisted sequential extraction (USE) is an alternative to the conventional BCR sequential extraction method (CSE). However, despite the usefulness and promising research results, the application of ultrasound waves for the extraction of heavy metals from sewage sludge is still very limited. Moreover, the studies conducted so far practically do not include municipal sewage sludges generated at different stages of processing, which differ not only in their content of heavy metals but also in their physicochemical properties [5,18,24,25,26]. One should consider that raw sludge from the beginning of the processing line, after drying, differs in structure from dewatered sludge. This means that each sample may require a different preparation procedure for the sequential extraction in order to avoid discrepancies caused by the effect of the different particle sizes of the analyzed material. This, in turn, may affect the quality of the obtained results despite maintaining the same extraction conditions.

The main aim of this study was to determine the content of Cd, Cr, Cu, Ni, Pb, Zn, and Hg in particular chemical fractions of sewage sludge generated at different stages of its processing (primary and dewatered sludge) using conventional and ultrasound-assisted BCR sequential extraction methods. In order to find out whether the different extraction approaches for heavy metal fractionation can reduce the total extraction time, the comparison of the obtained results was conducted. The reference material ERM-CC144 (Joint Research Center; JCR) was used to determine the main parameter of the ultrasound-assisted extraction method, i.e., the sonication time, and also for the internal check of the procedures used.

## 2. Materials and Methods

### 2.1. Material and Sampling

The research material consisted of two types of sewage sludge collected from one of the mechanical–biological wastewater treatment plants (WWTPs) located in Poland (Central Europe) that receives municipal and industrial wastewater. Sludge samples were collected at the initial (primary sludge) and final stage of the processing line (dewatered sludge). In order to be sure that samples were representative, each of them was collected by taking a few subsamples from various points in the same sampling site and then mixing them (about 3 kg of each sludge was collected). The primary sludge was sampled using a polypropylene baker, while the dewatered sludge was sampled with a shovel made of stainless steel. The different methods of sample collection resulted from differences in the consistency of the primary and dewatered sludge. The content of dry matter (DM) in the primary sludge reached 3%, while in the dewatered sludge, it reached 20%. This meant that during treatment, the sludge moisture content (MC) was reduced to 97% and 80%, respectively. After collection, all of the samples were kept in labeled polypropylene (PP) containers and stored in a refrigerator at 4 °C until further analysis.

### 2.2. Sample Preparation Procedure

**Figure 1:**
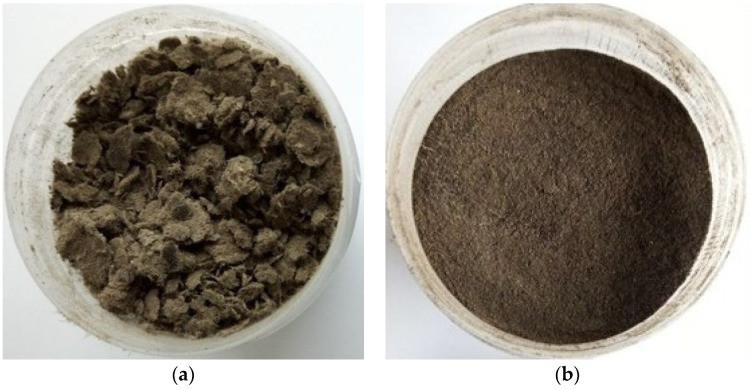
The (**a**) primary and (**b**) dewatered sludge after grinding and/or sieving.

The sludge samples required proper preparation before they were subjected to (a) mineralization (digestion) to determine the total content of heavy metals, as well as (b) chemical sequential extraction to determine their chemical forms of occurrence. The preparation of the sludge samples included drying in a laboratory dryer to a constant mass at 105 °C (SUP-100G, Wamed, Poland). Afterward, the dried sludge samples were milled in a mortar grinder (Fritsch Pulverisette 2 Mortar Grinder, Germany) and sieved through a 0.2 mm sieve. Due to the high moisture content and structure, the primary sludge required a longer drying time, and after its completion, it formed a thick and hard layer that was difficult to grind. Therefore, before grinding, the dried primary sludge was divided into very small particles with Teflon scissors. Despite this, the sludge particles were still too large to be sieved (Figure 1). Therefore, in this case, we omitted this step. After the completion of the preparation procedure, 0.2 g of the sludge sample was used for mineralization, and 0.5 g was used for chemical sequential extraction.

### 2.3. Determination of the Total Heavy Metal Content

The determination of the total heavy metal content must be preceded by the mineralization of the sample. For this purpose, 0.2 g of each of the sludge samples was digested with 5 mL 65% nitric acid (HNO_3_) and 15 mL 35–38% hydrochloric acid (HCl). Then, the mixture was placed in a Teflon flask and subjected to mineralization using a microwave digestion system (Multiwave 3000, Anton Paar GmbH, Graz, Austria). The mineralization program was as follows—first step: power 800 W, ramp 15 min, hold 5 min; second step: power 1400 W, ramp 15 min, hold 40 min; p-rate = 0.3 bar/s, IR = 240 °C (maximum temperature), and P_max_ = 60 bar (maximum power). After cooling, the obtained solutions were filtered through quantitative filter papers with a medium pore size (type 390) and diluted with 5% HNO_3_ to a volume of 50 mL. All of the sludge samples were stored at 4 °C until the laboratory analysis.

The total concentrations of heavy metals in the obtained solutions were determined using inductively coupled plasma optical spectrometry (Avio 200 ICP-OES, PerkinElmer Inc., Waltham, MA, USA). Only mercury was assayed with cold vapor atomic absorption spectrometry (CVAAS). The standards were prepared on the day of the analysis. The limits of detection (LODs) for the heavy metals were 0.004, 0.006, 0.005, 0.007, 0.009, 0.008, and 0.0001 mg·L^−^**^1^** for Cd, Cr, Cu, Ni, Pb, Zn, and Hg, respectively. The wavelengths for the analyzed elements were 214.440, 267.716, 324.752, 231.604, 220.353, 213.857, and 253.652 nm for Cd, Cr, Cu, Ni, Pb, Zn, and Hg, respectively.

### 2.4. Quality Control—Precision and Accuracy

The quality control was conducted using a certified reference material, ERM-CC144 (JRC). It was intended to check the precision and accuracy of the method used for the total heavy metal determination. The corrections to the dry mass were carried out according to the manufacturer’s instructions. The amount of sample used was 0.5 g. All of the microwave digestions were carried out in triplicate with a reagent blank. The recovery rates (R) for heavy metals in the reference material were between 87.4% and 101.0% (Equation (1)). Moreover, the average values of the relative standard deviation (RSD) were less than 10% for each of the analyzed elements, and ranged from 0.3% to 2.9% (Equation (2)). Therefore, the obtained results indicate that the conducted analysis was under control. The results of the heavy metal concentrations in the certified reference material are shown in Table 1.

(1) $$R=\frac{Total\;content\;of\;metal\;in\;sample\;}{Total\;content\;of\;metal\;in\;ERM-CC144}\times 100;\%\;$$

(2) $$RSD=\frac{Standard\;deviatation}{Mean}\times 100;\%$$

### 2.5. Chemical Reagents Used in the Study

A detailed list of the chemical reagents used in the BCR sequential extraction methods is presented in Table 2.

### 2.6. Sequential Extraction Methods

The three-stage conventional and ultrasound-assisted (modified) BCR sequential extraction methods were used for the fractionation of Cd, Cr, Cu, Ni, Pb, Zn, and Hg in two types of sewage sludge collected at the initial and final stage of the processing line. The presented paper takes into account heavy metals that are included in the Regulation of the Minister of the Environment of 6 February 2015 (J. L. 2015, Item. 257) (Poland) [27], as well as in the Council Directive of 12 June 1986 (86/278/EEC) (UE) [28].

The modified sequential extraction procedure requires a determination of the sonication time. The experiment was conducted using a certified reference material, ERM-CC144 (JCR). The choice of reference material depended on its availability and the range of the analyzed elements. Unfortunately, some of the commonly used materials, such as BCR-143R (JCR), BCR-144R (JCR) and BCR-146R (JCR), were unavailable (probably due to a coronavirus pandemic, COVID-19) and/or withdrawn from sale, while others, such as BCR-145R (JCR) or BCR-483 (JCR), did not include some of the analyzed elements.

#### 2.6.1. Conventional Chemical Sequential Extraction Method

The conventional BCR sequential extraction method includes three main stages and one additional, which is optional [12,13,20]. The characteristics of the individual stages of the sequential extraction are presented below. 

In Stage I, the extraction of the exchangeable fraction, that is, the fraction bound to carbonates (F1; mobile), 20 mL 0.11 M acetic acid (CH_3_COOH) was added to 0.5 g of the dried sludge sample, transferred to a centrifuge tube, and shaken for 16 h at room temperature (at 130 rpm; GFL 30116, Germany). Afterward, the extract was spun in a laboratory centrifuge for 5 min (at 20,000 rpm; Avanti JXN-26, Beckman Coulter, USA), while the supernatant was poured into a polyethylene container and left for analysis. The residue was washed with 10 mL deionized water (HLP 10UV, Hydrolab, Poland; the water met the parameters of PN-EN 3696:1999 [29], I degree of cleanliness), then shaken for 15 min, and configured. The supernatant was discarded. 

In Stage II, the extraction of the reducible fraction, that is, the fraction bound to Fe/Mn oxides (F2; mobile), 20 mL 0.1 M hydroxylamine hydrochloride (NH_2_OH·HCl), adjusted to pH = 2 with nitric acid (HNO_3_), was added to the residue sludge from Stage I. The subsequent stages were carried out following the procedure in Stage I.

In Stage III, the extraction of the oxidizable fraction, that is, the fraction bound to organic matter and sulfides (F3; immobile), 5 mL of 8.8 M hydrogen peroxide (H_2_O_2_) was added to the residue from Stage II. After that, the sample was incubated at 85 °C for 1 h in a laboratory water bath. The above procedure was repeated. Afterward, 25 mL of 1 M ammonium acetate (CH_3_COONH_4_), adjusted to pH = 2 with HNO_3_, was added. The subsequent steps were carried out following the procedure in Stage I and II. 

In Stage IV, the extraction of the residual fraction with aqua regia (F4; immobile), 5 mL nitric acid (HNO_3_) and 15 mL hydrochloric acid (HCl) were added to the residue from Stage III. The mixture was subjected to mineralization using a microwave digestion system (Multiwave 3000, Anton Paar GmbH, Graz, Austria), as in the case of the dry sludge samples.

The concentrations of heavy metals in the obtained extracts were determined using inductively coupled plasma optical spectrometry (Avio 200 ICP-OES, PerkinElmer Inc., Waltham, MA, USA). Only mercury was assayed with cold vapor atomic absorption spectrometry (CVAAS). All of the analyses were repeated twice.

For the internal check of the applied procedure and the verification of the obtained results, we calculated the recovery of the method (R_M_) by comparing the sum of the four chemical fractions (SF) with the total concentrations of heavy metals (TMC) in the certified reference material (Equation (3)) [20]. We applied this formula both for the conventional and modified sequential method. The same procedure was used for primary and dewatered sewage sludge.

(3) $$R_{M}=\frac{SF}{TMC}\times 100;\%$$

#### 2.6.2. Ultrasound-Assisted Chemical Sequential Extraction Method

As previously mentioned, the modified chemical sequential extraction procedure requires the determination of the sonication time, which will reduce the total time of the extraction, and at the same time will provide results similar to those obtained with the conventional method. Optimization studies were carried out using the certified reference material, ERM-CC144 (JCR), which was obtained from sewage sludge of domestic origin.

**Figure 2:**
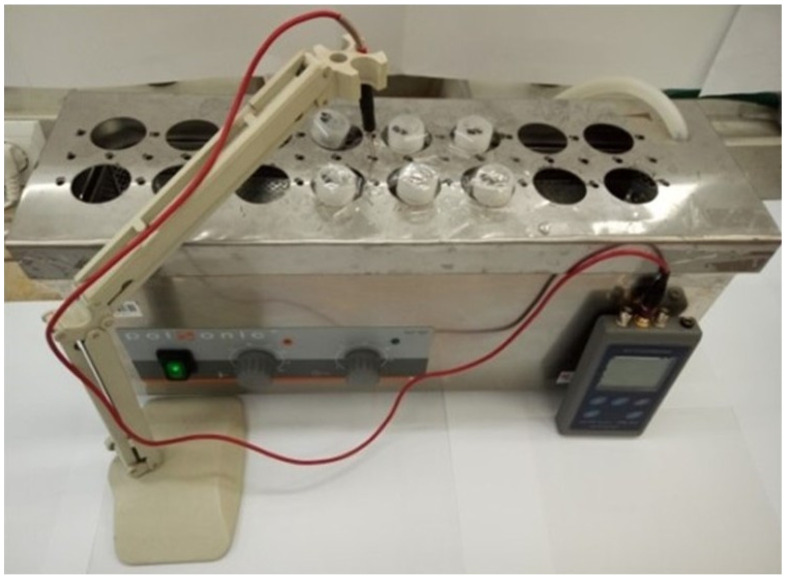
Laboratory-scale setup for the ultrasound treatment, equipped with temperature and time controllers.

The ultrasound extraction was carried out in an ultrasonic bath (Sonic-5; Polsonic; Poland) with a 6 L capacity, equipped with time and temperature controllers. This device enabled the treatment of many samples simultaneously. The working parameters of the ultrasonic bath used in the discussed experiment were 40 kHz (frequency) and 2 × 320 W (power). The temperature during the experiment was fixed at 30 ± 5 °C. Similar temperatures have been observed in relation to the samples after shaking for 16 h. The laboratory setup for sonication is presented in Figure 2.

In our experiment, we tested a sonication time in the range of 10 min to 60 min. All of the analyses were performed in duplicate. In order to make the reference material mix well with the individual reagents used in the extraction procedure, the mixture was shaken for 30 min (130 rpm) at room temperature before sonication (in Stages I and II). All of the remaining activities associated with the extraction process were carried out following the conventional procedure. A comparison of the conventional [12,13] and ultrasound-assisted BCR extraction methods is shown in Table 3.

In order to compare the results obtained for the two tested extraction methods, we calculated the percentage differences, both in relation to ERM-CC144 (JCR) and the two sewage sludges collected from the processing line (Equation (4)) [30].

(4) $$\%\;difference=\frac{\left|V_{1}-V_{2}\right|}{\left|\frac{V_{1}+V_{2}}{2}\right|}\times 100;\;\%$$

where *V*_1_ and *V*_2_ are the values of the metal content in the tested sample extracted by the conventional and ultrasound-assisted methods, respectively.

## 3. Results and Discussion

### 3.1. The Effect of the Sonication Time on the Extraction of Heavy Metals

**Figure 3:**
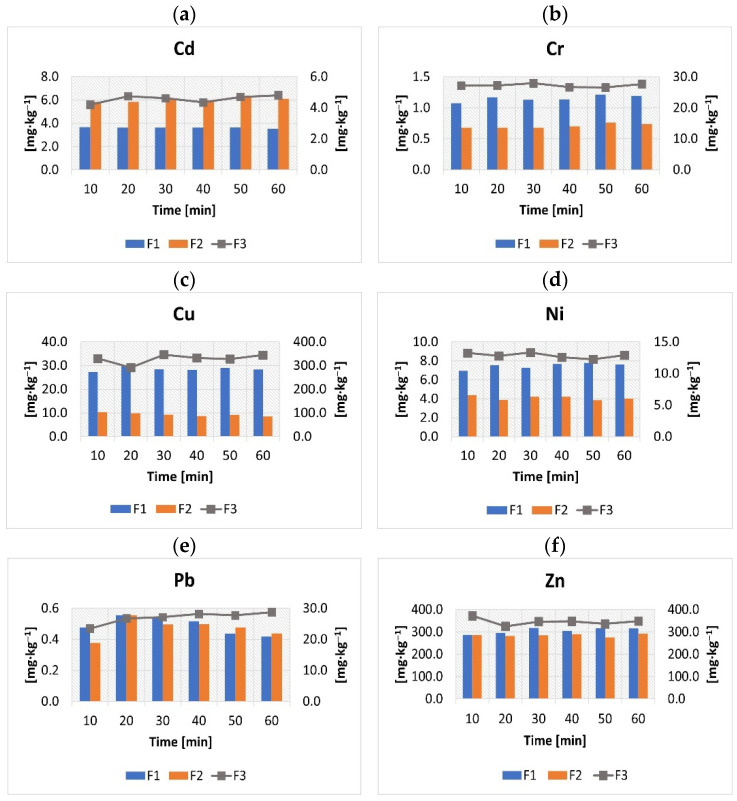
(**a**–**f**) The effects of the sonication time on the extraction of heavy metals in the three main fractions of the ultrasound-assisted BCR method.

The effect of the sonication treatment time on the extraction of heavy metals in the three main fractions of the ultrasound-assisted BCR method is shown in Figure 3. We do not present the results obtained for mercury because this element was found only in the first and the residual fraction.

The conducted experiment shows that in the case under consideration, the effects obtained for different sonication times did not significantly differ (Figure 3). This could be due to the relatively high power of the ultrasonic bath used in the experiment. However, it can be noticed that the content of the analyzed metals in the three main chemical fractions increases slightly after 10 to 30 min, e.g., in the case of copper, lead, and zinc. Taking into account the purpose of these studies and the different characteristics of the sewage sludge generated in the technological line of the WWTP, we decided that 30 min would be the optimal time for sonication. Moreover, previous studies by Kazi et al. (2006) [18], who attempted to determine the optimal time of sonication by using the BCR-483 reference material, indicate the correctness of the adopted assumptions. Thanks to the use of ultrasound waves, the time of the first three stages was shortened to 4 h and 30 min (excluding configuration, filtration, and the washing of the sample). Research on the effect of the sonication time on the share of heavy metals in different chemical fractions of sewage sludge was also conducted by other scientists. These studies showed that the obtained results vary depending on the type of ultrasound processor, and its power and frequency [18,24,25,26]. Therefore, in the discussed experiment, for ultrasound treatment, an ultrasonic bath with typical work parameters (frequency and power) was used. Moreover, as was previously mentioned, the temperature during the process was kept at a constant level.

### 3.2. Comparison of the Conventional and Ultrasound-Assisted BCR Sequential Extraction

Following scientific literature reports [18,19,26], the ultrasound-assisted BCR sequential extraction method based on the use of ultrasound waves for heavy metal extraction allows similar results in comparison with the conventional BCR extraction method to be obtained. However, the extraction efficiency of heavy metals from solid samples like sewage sludge depends on several factors and technical parameters of the process, i.e., the type of ultrasound processor, the frequency and intensity characteristics of the wave, the sonication time, and the temperature [25,26,31]. Therefore, the optimization of different factors that have an impact on the process of ultrasound-assisted BCR sequential extraction is important in order to obtain results which are comparable to those achieved by the conventional method. Each of these factors should be tested separately. Moreover, it must be emphasized that during ultrasound treatment, some properties of the sewage sludge may change, which as a result may lead to different fractionation patterns in comparison to classical shaking.

However, taking into account the number of scientific papers referencing the use of ultrasound waves in the process of the sequential extraction of heavy metals from soils or sediments [19,23,31,32,33,34,35,36], there are still only a few studies that concern sewage sludge [5,18,24,25,26]. Therefore, we decided to undertake research in this direction.

#### 3.2.1. Heavy Metals in the ERM-CC144 (JRC)

The comparison results of the conventional and ultrasound-assisted sequential extraction method for ERM-CC144 (JRC) certified material are presented in Table 4 and Table 5.

We used the ERM-CC144 (JRC) only to determine the sonication time and internal check of the procedures used. The conducted experiment revealed that almost all of the analyzed heavy metals in ERM-CC144 (JCR) were characterized by a high recovery rate (R_M_), with the only exception being mercury. Presumably, this was related to its volatility and low concentration in the examined samples, which was also observed in our previous studies [6,10,20]. The overall recoveries of Cd, Cr, Cu, Ni, Pb, Zn, and Hg obtained by using the ultrasonic bath at 30 min were 121.6%, 94.6%, 113.8%, 94.8%, 96.5%, 105.2%, and 24.8%, respectively. In comparison, by using the conventional shaking, the recoveries were 123%, 90.9%, 111.0%, 92.0%, 97.4%, 105.1%, and 24.9%, respectively. Therefore, it can be concluded that the recovery values obtained by the ultrasound-assisted extraction are close to those achieved by the conventional method. Similar results were previously reported by other scientists, who used ultrasound-assisted extraction to release the same heavy metals from the BCR-483 certified reference material, and achieved recovery levels of 91.6% to 99.9% [18]. However, the conducted experiment revealed the difference in the fractionation pattern of Zn. The mean content of zinc in different chemical fractions of ERM-CC144 (JRC) followed the orders F1 > F2 > F3 > F4 and F1 > F3 > F2 > F4 for the conventional and ultrasound-assisted extraction methods, respectively. Despite this, the percentage shares of this element in the sum of mobile fractions for both methods were still the highest.

In order to compare the content of heavy metals extracted from ERM-CC144 (JCR) following the conventional and ultrasound-assisted methods, the percent differences were calculated (Table 4), with the only exception being mercury. This is because the recoveries obtained for this element in both procedures of sequential extraction were unsatisfactory. The conducted calculations showed that the percentage differences between the two tested methods were in the range of 0.1% to 4.0%, which confirms that ultrasound treatment does not significantly affect the obtained results, and can be used in further research.

Moreover, it is worth noting that our research provides additional data on ERM-CC144 (JCR). The brochure of this certified reference material did not include values for the heavy metal content at each stage of the BCR sequential extraction so far. Therefore, the obtained results may be a contribution to conducting further research in this area, and they complement the missing data in the ERM-CC144 (JCR) material certificate, which is an additional advantage of the discussed research.

#### 3.2.2. Heavy Metals in the Primary and Dewatered Sewage Sludge

In the scientific literature, the effects of ultrasound-assisted BCR sequential extraction differ for soils, sediments, and sewage sludge, etc. [5,19,37]. Moreover, the recovery values for particular heavy metals are not always comparable with those obtained by the conventional method. Considering solid samples, there are very few studies in the literature investigating the influence of ultrasound treatment on the extraction of heavy metals from sewage sludge. So far, the best results were obtained by Pérez at al. (1998) [24] and Kazi et al. (2006) [18], who achieved recoveries in the range of 90.0 to 117% (Cu, Cr, Ni, Pb, Zn) and 96.0 to 99.6% (Cd, Cr, Cu, Ni, Pb, Zn), respectively.

The total contents of seven heavy metals in the analyzed primary and dewatered sewage sludge ranged from 0.49 mg·kg^−1^ to 696.62 mg·kg^−1^ and 2.51 mg·kg^−1^ to 1026.90 mg·kg^−1^, respectively (Table 6). The average concentrations of these elements ranked in the following order: Zn > Cu > Ni > Cr > Pb > Cd > Hg. According to the literature data, most often, the concentrations of particular heavy metals in sewage sludge can be ordered as follows: Zn > Cu > Pb > Ni > Cr > Cd [18]; Zn > Cu > Pb > Cr > Ni > Cd > Hg [6], or Zn > Cu > Cr > Ni > Pb > Cd [2], etc. Therefore, we can proclaim that the composition of the analyzed sewage sludge is typical for municipal WWTPs.

The comparison results of the conventional and ultrasound-assisted sequential extraction methods for primary and dewatered sewage sludge are presented in Table 6, Table 7 and Table 8. The verification of the two sequential extraction methods was conducted using the recovery rate (R_M_). In relation to the concentrations of Cd, Cr, Cu, Ni, Pb, Zn, and Hg, in the primary sludge, the recoveries were in the range of 62.8–130.2% and 79.8–135.7% for the conventional and modified extraction method, respectively, whereas for dewatered sludge, the recoveries were in the range of 87.2–105.7% and 87.8–112.2%. The obtained results seem to be comparable. The only exception was Hg in dewatered sludge (8.7% and 14.4%, respectively). As we previously mentioned, presumably, this was related to its volatility in the examined samples. The calculations also revealed that high recovery values (over 130%) were recorded in relation to cadmium in the primary sludge. This may be due to several factors. Considering that dried primary sludge is difficult to grind and sieve due to its structure, a possible reason for the lower release of cadmium from the analyzed sample was its insufficient mineralization or preparation. Presumably, it will be necessary to increase the temperature or extend the mineralization time in order to improve the process efficiency. It is also possible that instead of dividing it into very small particles with Teflon scissors, it would be more appropriate to freeze it (preceded by drying), grind it, and then dry it again. Another reason may be the LOD. Taking into account that cadmium is present in sewage sludge at low concentrations, it should perhaps be necessary to perform the analysis using inductively coupled plasma mass spectrometry (ICP-MS) instead of inductively coupled plasma optical spectrometry (ICP-OES), which is characterized by a greater measurement accuracy. However, further research is needed in order to investigate this issue in more detail.

Table 7 and Table 8 present the percentage differences between the content of heavy metals in both tested methods of sequential extraction. The calculations indicated that the content of analyzed heavy metals (except Hg) in the four chemical fractions was in the range of 0.8% to 14.6% and 0.8% to 8.7% for the conventional and ultrasound-assisted extraction methods, respectively. The greatest differences were indicated in relation to Cr, Cu, and Pb extracted from the primary sludge with the conventional method. However, regardless of the sewage sludge type, it was found that higher concentrations of analyzed heavy metals (in total) were obtained by using the modified sequential extraction method (the only exception in both types of sludge was Zn, along with Ni in the dewatered sludge). This is mainly because in some cases, in the samples subjected to ultrasound treatment, the amount of heavy metals released was higher. Presumably, this is due to the effect of the cavitation phenomenon on the sample’s material structure, which caused an increase of the sludge particles’ specific surface area, and in turn resulted in the better mixing of the sample with the extractants in comparison with the conventional shaking. The above findings confirmed those of another researcher, who achieved a higher content of Cu, Pb, and Zn in the BCR-141R certified reference material (calcareous loam soil) as a result of the ultrasound treatment application. However, at the same time, they also indicate that for the BCR-701 and BCR-601 reference materials (lake sediments), higher concentrations of Cd, Cr, Cu, Ni, Pb, and Zn were obtained by the conventional sequential extraction [37]. Similar observations made by other scientists indicate that the use of ultrasound waves during the sequential extraction process promotes the increased release of Cd and Pb but, at the same time, decreases the Cu and Zn concentrations [5].

**Figure 4:**
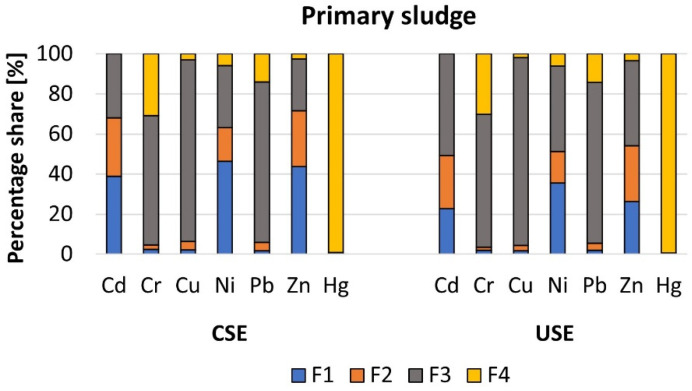
Percentage share of heavy metals in different chemical fractions of primary sludge.

The mean content of heavy metals in different chemical fractions of primary sludge followed the orders F1 > F3 > F2 > F4 and F3 > F2 > F1 > F4 (Cd), F3 > F4 > F1 > F2 (Cr), F3 > F2 > F4 > F1 (Cu), F1 > F3 > F2 > F4 and F3 > F1 > F2 > F4 (Ni), F3 > F4 > F2 > F1 (Pb), F1 > F2 > F3 > F4, and F3 > F2 > F1 > F4 (Zn), F4 > F1 > F2 = F3 (Hg) for the conventional and ultrasound-assisted extraction methods, respectively (Figure 4). The different fractionation patterns in comparison with conventional extraction were found in relation to Cd, Ni, and Zn. The percentage share of these elements in the mobile fractions (F1 and F2) was as follows: 68.1% and 49.3% (Cd); 63.3% and 51.3% (Ni); and 71.6% and 54.1% (Zn) for the conventional and ultrasound-assisted extraction methods, respectively. In all cases, the share of metals in the mobile fractions, especially in the fraction F1, decreased in favor of the fraction F3. In relation to some heavy metals, the percentage differences in the content of a given metal in the individual fractions were very small, as in the case of zinc (CSE-F2 and F3; USE-F1 and F2). Despite the observed differences, the total content of heavy metals in the sum of all of the chemical fractions was similar for both methods. The decrease in the Cd and Ni concentrations in the mobile fractions was also noted by other researchers, who used the ultrasound-assisted method to extract heavy metals from the BCR-701 certified material (lake sediments) [19]. Similar observations with regard to Zn were made by other scientists, who found differences between the concentration of this element in mobile fractions of raw and clarified sewage sludge, i.e., 119.1 mg·kg^−1^ and 140.0 mg·kg^−1^ for the conventional method and 1714.7 mg·kg^−1^ and 1168.0 mg·kg^−1^ for the ultrasound-assisted extraction method, respectively [5]. Unfortunately, the sources of the errors influencing the obtained results were still not indicated.

In conclusion, the differences in the fractionation patterns of the individual heavy metals in this study may result from the type of sewage sludge and its characteristics. As we previously mentioned, primary sludge is highly hydrated and difficult to dry and grind. This may influence its mixing with individual extractants. It is, therefore, necessary to expect that in this case it will be necessary to extend the time of shaking before the first two stages of extraction for the proper mixing of the primary sludge with the extractants. Moreover, as previously mentioned, the extension of the sample preparation procedure for extraction may also be required. However, in this case, further research is needed in order to develop an appropriate sludge preparation technique, and to optimize the process.

**Figure 5:**
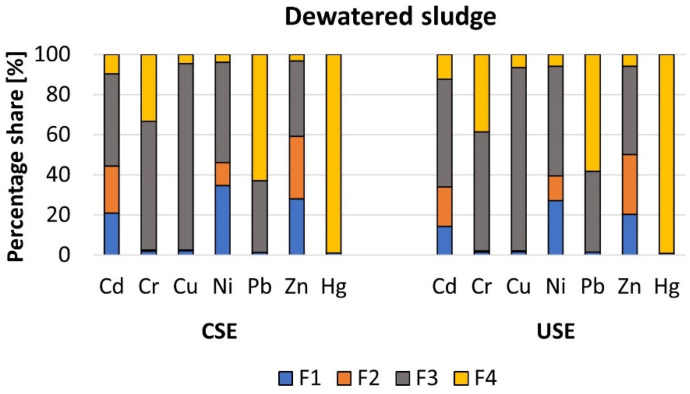
Percentage share of heavy metals in the different chemical fractions of dewatered sludge.

The mean content of analyzed heavy metals in different chemical fractions of dewatered sludge was identical for both extraction methods, and followed the orders F3 > F2 > F1 > F4 (Cd), F3 > F4 > F1 > F2 (Cr), F3 > F4 > F1 > F2 (Cu), F3 > F1 > F2 > F4 (Ni), F4 > F3 > F1 > F2 (Pb), F3 > F2 > F1 > F4 (Zn), and F4 > F1 > F2 = F3 (Hg), respectively (Figure 5). The most visible differences between the concentrations of analyzed HMs in the mobile fractions, in comparison to those achieved by the conventional sequential extraction method, were noted in relation to Cd, Ni, and Zn. A similar tendency (in relation to Zn) was also stated in the ERM-CC144 (JCR) certified material. The above findings were also observed by other scientists [18,24]. The percentage shares of Cd, Ni, and Zn in the mobile fractions (F1 and F2) were as follows: 44.4% and 34.0% (Cd), 46.2% and 39.4% (Ni), and 59.3% and 50.0% (Zn) for the conventional and ultrasound-assisted extraction methods, respectively. On the other hand, in total, the content of heavy metals in the sum of the four chemical fractions was similar for both procedures. Therefore, it can be stated that an ultrasound-assisted sequential extraction method may be an alternative to the conventional method.

In order to check whether the differences between the concentrations or fractionation patterns of particular heavy metals are significant, we decided to assess the potential ecological risk that these elements may pose to the natural environment and living organisms. For this purpose, the values of two selected indices were calculated, namely the Risk Assessment Code (RAC) [38] and Individual Contamination Factor (ICF) [39,40]. This is essential in the case of the natural use of sewage sludge, especially the dewatered sludge (the final sludge). The first index presents the percentage share of a particular metal in the first, most mobile chemical fraction (no risk 1% ≥ RAC > 50.0% very high risk), while the second index is the value of the quotient of the sum of the metal amount in fractions from F1 to F3, and the amount of metal in fraction F4, i.e. ICF = (F1 + F2 + F3)/F4 (low contamination 1 ≥ ICF > 6 very high contamination).

The RAC values calculated for Cd, Ni, and Zn in the primary sludge indicated that these elements may pose high/moderate (CSE 38.9% and USE 22.8%), high (CSE 46.4% and USE 35.7%), and high/moderate risk (CSE 43.9% and USE 26.3%). In turn, the ICF values revealed that the primary sludge is very highly contaminated with Ni (CSE 16.4; USE 15.6) and Zn (CSE 40.8; USE 28.5). In the case of Cd, it was impossible to make the necessary calculations due to the fact that the concentration of this metal in the fourth chemical fraction equals 0. Despite minor differences in the interpretation of the level of ecological risk, in relation to primary sludge, it should be remembered that fraction F1 is not the only mobile fraction. Moreover, the RAC index does not fully reflect the level of the risk because it does not includes the second mobile fraction, i.e., F2. Moreover, in contrast to RAC, the values of the ICF index confirmed that the minor differences between the two tested methods have no significant impact on the interpretation of the ecological risk or contamination level, regardless of the sewage sludge type.

As we previously mentioned, in the case of dewatered sewage sludge, no difference was found regarding the heavy metal fractionation patterns, but we noted lower concentrations of Cd, Ni, and Zn in the mobile fractions extracted by the ultrasound-assisted extraction method. Therefore, in this case, we also calculated the values of RAC and ICF for these elements. The obtained results indicate that Cd, Ni, and Zn pose a moderate (CSE 21.0% and USE 14.4%), high/moderate (CSE 34.6% and USE 27.0%), and moderate (CSE 28.0% and USE 20.2%) potential ecological risk; additionally, the dewatered sludge is very highly polluted with all three heavy metals regardless of the method used, i.e., 9.3 (CSE) and 8.1 (USE), 24.5 (CSE) and 15.8 (USE), and 30.8 (CSE) and 16.1 (USE), respectively.

Therefore, it can be stated that despite the differences in the concentrations or fractionation patterns of the particular heavy metals in particular chemical fractions, the results obtained by the ultrasound-assisted sequential extraction method are valid and do not significantly affect the potential ecological risk assessment. However, it is necessary to conduct further research aimed at developing a methodology for the preparation of primary sludge for the extraction process. Moreover, it would be valuable to develop a new ecological risk index that takes into account the contribution of heavy metals in both mobile fractions (F1 and F2).

## 4. Conclusions

The conducted research was aimed at the determination of the concentrations of Cd, Cr, Cu, Ni, Pb, Zn, and Hg in particular chemical fractions of the primary and dewatered sewage sludge originating from the municipal WWTP. The chemical fractions were obtained by conventional and ultrasound-assisted BCR sequential extraction methods. The experiment revealed that the most optimal sonication time for sequential extraction is 30 min. Moreover, it was demonstrated that ultrasound treatment allows the reduction of the extraction time to 4 h and 30 min (stages I–III), which reduces energy consumption.

The recoveries of heavy metals ranged from 62.8% to 130.2% (CSE) and 79.8% to 135.7% (USE), and 87.2% to 113.2% (CSE) and 87.8% to 112.0% (USE), for primary and dewatered sludge, respectively. The only exception was mercury, of which the recoveries in both methods were insufficient. It was shown that, regardless of the sludge type, the greatest share in the mobile fractions (F1 and F2) was taken by Cd, Ni and Zn. For the primary sludge, the percentage share of these elements in the sum of factions F1 and F2 was as follows: 68.1% and 49.3% (Cd), 63.3% and 51.3% (Ni), and 71.6% and 54.1% (Zn), for the conventional and ultrasound-assisted extraction methods, respectively. On the other hand, for the dewatered sludge, the results were as follows: 44.4% and 34.0% (Cd), 46.2% and 39.4% (Ni), 59.3% and 50.0% (Zn), respectively. The conducted research showed that further research must be conducted regarding primary sludge preparation before extraction. It was revealed that, due to its characteristics, the differences in the fractionation patterns of Cd, Ni and Zn in comparison to the conventional extraction method were observed. Moreover, minor differences in the concentrations of Cd, Ni and Zn in the chemical fractions of dewatered sludge were revealed. However, despite all of the above findings, it was confirmed that, in general, they do not significantly influence the results of the ecological risk assessment (except for Cd and Zn in the primary sludge). This means that the ultrasound-assisted extraction method can be considered as a “green method” for the assessment of the bioavailability and mobility of heavy metals in solid samples.

In conclusion, the obtained results indicate that ultrasound-assisted sequential extraction can be an effective alternative to the conventional method for the fractionation of heavy metals in sewage sludge.

## Figures and Tables

**Table 1:** Heavy metal concentrations in the ERM-CC144 (JRC) certified reference material.

Heavy Metal	This Study	ERM-CC144	R	RSD
	mg·kg^−1^	mg·kg^−1^	%	%
Cd	12.67 ± 0.18	14.5	87.4	1.4
Cr	151.23 ± 0.52	168.0	90.0	0.3
Cu	351.60 ± 2.58	348.0	101.0	0.7
Ni	83.51 ± 0.23	91.0	91.8	0.3
Pb	151.60 ± 1.64	157.0	96.6	1.1
Zn	927.58 ± 9.02	980.0	94.7	1.0
Hg	5.16 ± 0.15	5.9	87.4	2.9

Results are expressed as the mean ($\bar{x}$) ± standard deviation (SD) in mg·kg^−1^ of dry matter (DM).

**Table 2:** List of chemical reagents used in the study.

Chemical Reagent	Chemical Formula	Purity	Commercial Brand	Country of Origin
Acetic acid (V); 65%	HNO_3_	analysis-pure (a.p.)	POCH	Poland
Hydrochloric acid; 35–38%	HCl	analysis-pure (a.p.)	POCH	Poland
Acetic acid; 99.5–99.9%	CH_3_COOH	analysis-pure (a.p.)	POCH	Poland
Hydroxylamine hydrochloride	NH_2_OH·HCl	analysis-pure (a.p.)	Chempur	Poland
Hydrogen peroxide; 30%	H_2_O_2_	analysis-pure (a.p.)	Chempur	Poland

All of the data in Table 2 are given in accordance with manufacturers’ specifications.

**Table 3:** A comparison of the conventional and ultrasound-assisted BCR sequential extraction methods [12,13] (this study).

Fraction	Extraction Agent	Conventional BCR (CSE)	Ultrasound-Assisted BCR (USE)
		Extraction Time	
Acid soluble/exchangeable fraction; bound to carbonates (F1)	20 mL CH_3_COOH (0.11 M)	Shake for 16 h	Shake for 30 min and sonicate for 30 min
Reducible fraction; bound to Mn and Fe oxides (F2)	20 mL NH_2_OH·HCl (0.1 M, pH = 2)	Shake for 16 h	Shake for 30 min and sonicate for 30 min
Oxidizable fraction; bound to organic matter and sulfides (F3)	5 mL H_2_O_2_ (8.8 M, pH = 2), heat to 85 °C for 1 h (repeat twice); 25 mL CH_3_COONH_4_ (1 M, pH = 2)	Shake for 16 h	Sonicate for 30 min
Residual fraction (F4)	15 mL HCl/5 mL HNO_3_ (3:1) (aqua regia; microwave digestion).	-	-

**Table 4:** Comparison of the results obtained by the conventional and ultrasound-assisted sequential extraction methods based on the heavy metal content in ERM-CC144 (JCR).

Fraction	HM	Conventional Extraction (CSE)	Ultrasound-Assisted Extraction (USE)
		mg·kg^−1^	
F1	Cd	3.69 ± 0.12	3.63 ± 0.14
F2		6.96 ± 0.17	6.06 ± 0.26
F3		4.03 ± 0.13	4.62 ± 0.35
F4		0.90 ± 0.10	1.09 ± 0.00
Total content		12.67 ± 0.18	
RM; %		123.0	121.6
F1	Cr	1.29 ± 0.02	1.13 ± 0.03
F2		0.84 ± 0.00	0.68 ± 0.00
F3		28.18 ± 0.28	27.92 ± 0.92
F4		107.17 ± 4.61	113.37 ± 12.45
Total content		151.23 ± 0.52	
RM; %		90.9	94.6
F1	Cu	31.93 ± 0.90	28.43 ± 0.43
F2		10.17 ± 0.33	9.27 ± 0.54
F3		334.24 ± 5.70	346.14 ± 1.32
F4		13.83 ± 0.40	16.33 ±0.22
Total content		351.60 ± 2.58	
RM; %		111.0	113.8
F1	Ni	8.75 ± 0.24	7.29 ± 0.02
F2		5.23 ± 0.10	4.21 ± 0.28
F3		10.31 ± 0.27	13.33 ± 0.26
F4		52.56 ± 1.66	54.35 ± 5.59
Total content		83.51 ± 0.23	
RM; %		92.0	94.8
F1	Pb	0.38 ± 0.02	0.54 ± 0.03
F2		0.40 ± 0.07	0.50 ± 0.03
F3		41.05 ± 0.82	27.15 ± 2.36
F4		105.78 ± 1.79	118.18 ± 9.72
Total content		151.60 ± 1.64	
RM; %		97.4	96.5
F1	Zn	395.90 ± 3.10	318.16 ± 3.61
F2		357.49 ± 6.99	284.35 ± 3.27
F3		203.90 ± 3.63	346.24 ± 3.45
F4		17.67±1.25	27.30 ± 0.54
Total content		927.58 ± 9.02	
RM; %		105.1	105.2
F1	Hg	0.003 ± 0.00	0.002 ± 0.00
F2		0.000 ± 0.00	0.000 ± 0.00
F3		0.000 ± 0.00	0.000 ± 0.00
F4		1.282 ± 0.20	1.279 ± 0.20
Total content		5.16 ± 0.15	
RM; %		24.9	24.8

Results are expressed as the mean ($\bar{x}$) ± standard deviation (SD) in mg·kg^−1^ of dry matter (DM).

**Table 5:** The percentage differences between the content of heavy metals in the two methods of sequential extraction in ERM-CC144 (JCR).

HM	Sum of the 4 Chemical Fractions		Percent Difference between CSE and USE
	Conventional Extraction (CSE)	Ultrasound-Assisted Extraction (USE)	
	mg·kg^−1^		%
Cd	15.57 ± 0.23	15.40 ± 0.23	1.1
Cr	137.48 ± 4.44	143.10 ± 11.50	4.0
Cu	390.17 ± 6.46	400.17 ± 0.98	2.5
Ni	76.85 ± 1.91	79.17 ± 6.11	3.0
Pb	147.61 ± 2.08	146.36 ± 7.41	0.9
Zn	974.97 ± 8.80	976.10 ± 3.96	0.1

Results are expressed as the mean ($\bar{x}$) ± standard deviation (SD) in mg·kg^−1^ of dry matter (DM).

**Table 6:** Comparison of the results obtained by the conventional and ultrasound-assisted sequential extraction methods based on the heavy metal content in the primary and dewatered sludge.

Fraction	HM	Conventional Extraction (CSE)		Ultrasound-Assisted Extraction (USE)	
		Primary Sludge	Dewatered Sludge	Primary Sludge	Dewatered Sludge
		mg·kg^−1^		mg·kg^−1^	
F1	Cd	1.95 ± 0.08	0.97 ± 0.06	1.19 ± 0.07	0.70 ± 0.02
F2		1.46 ± 0.02	1.08 ± 0.04	1.38 ± 0.06	0.96 ± 0.06
F3		1.59 ± 0.01	2.11 ± 0.14	2.64 ± 0.07	2.62 ± 0.16
F4		0.00 ± 0.00	0.45 ± 0.07	0.00 ± 0.00	0.60 ± 0.14
Total content		3.84 ± 0.15	4.35 ± 0.16	3.84 ± 0.15	4.35 ± 0.16
RM; %		130.2	105.7	135.7	112.2
F1	Cr	1.22 ± 0.04	1.44 ± 0.04	1.16 ± 0.02	1.16 ± 0.02
F2		1.21 ± 0.02	0.41 ± 0.02	0.96 ± 0.04	0.41 ± 0.02
F3		33.22 ± 1.09	46.85 ± 1.39	39.23 ± 0.94	45.14 ± 1.01
F4		15.82 ± 0.70	24.33 ± 1.90	17.86 ± 0.20	29.33 ± 0.91
Total content		53.17 ± 1.39	72.17 ± 1.35	53.17 ± 1.39	72.17 ± 1.35
RM; %		96.8	101.2	111.4	105.4
F1	Cu	6.25 ± 0.24	5.15 ± 0.10	5.73 ± 0.16	4.11 ± 0.25
F2		11.11 ± 0.66	0.97 ± 0.06	8.43 ± 0.54	0.86 ± 0.06
F3		246.74 ± 6.00	226.11 ± 10.94	294.97 ± 2.52	224.24 ± 3.86
F4		8.16 ± 1.08	10.92 ± 0.05	5.98 ± 0.19	15.78 ± 0.41
Total content		281.85 ± 14.12	278.94 ± 4.21	281.85 ± 14.12	278.94 ± 4.21
RM; %		96.6	87.2	111.8	87.8
F1	Ni	36.83 ± 0.56	54.54 ± 0.48	28.55 ± 0.50	41.4 3± 0.78
F2		13.43 ± 0.13	18.34 ± 0.97	12.51 ± 0.15	18.95 ± 0.84
F3		24.60 ± 2.81	78.60 ± 2.36	34.18 ± 0.28	83.76 ± 4.45
F4		4.58 ± 0.40	6.17 ± 0.55	4.81 ± 0.06	9.13 ± 0.47
Total content		73.18 ± 2.98	150.58 ± 2.04	73.18 ± 2.98	150.58 ± 2.04
RM; %		108.5	104.7	109.4	101.8
F1	Pb	0.74 ± 0.06	0.71 ± 0.17	0.95 ± 0.18	1.06 ± 0.09
F2		1.82 ± 0.13	0.00 ±0.00	1.66 ± 0.12	0.00 ± 0.00
F3		33.97 ± 0.65	22.30 ± 0.68	38.64 ± 0.99	27.14 ± 0.86
F4		5.94 ± 0.41	38.93 ± 2.71	6.87 ± 0.36	38.94 ± 0.86
Total content		45.04 ± 2.09	67.14 ± 2.60	45.04 ± 2.09	67.14 ± 2.60
RM; %		94.3	92.3	106.8	100.7
F1	Zn	356.35 ± 2.67	325.17 ± 2.69	201.53 ± 3.24	224.72 ± 6.02
F2		225.28 ± 4.17	363.49 ± 7.16	214.12 ± 5.69	331.61 ± 23.06
F3		210.91 ± 1.31	436.95 ± 3.66	325.92 ± 4.17	490.79 ± 8.03
F4		19.44 ± 0.35	36.58 ± 2.22	26.03 ± 0.60	65.24 ± 3.34
Total content		696.62 ± 17.01	1026.90 ± 5.03	696.62 ± 17.01	1026.90 ± 5.03
RM; %		116.6	113.2	110.2	108.3
F1	Hg	0.003 ± 0.01	0.003 ± 0.01	0.002 ± 0.01	0.003 ± 0.02
F2		0.000 ± 0.00	0.000± 0.00	0.000 ± 0.00	0.000 ± 0.00
F3		0.000 ± 0.00	0.000 ± 0.00	0.000 ± 0.00	0.000 ± 0.00
F4		0.303 ± 0.02	0.216 ± 0.01	0.387 ± 0.02	0.357 ± 0.03
Total content		0.49 ± 0.10	2.51 ± 0.05	0.49 ± 0.10	2.51 ± 0.05
RM; %		62.8	8.7	79.8	14.4

Results are expressed as the mean ($\bar{x}$) ± standard deviations (SD) in mg·kg^−1^ of dry matter (DM).

**Table 7:** The percentage differences between the content of heavy metals in the two methods of sequential extraction in primary sewage sludge.

HM	Sum of the 4 Chemical Fractions		Percent Difference between CSE and USE
	Conventional Extraction (CSE)	Ultrasound-Assisted Extraction (USE)	
	mg·kg^−1^		%
Cd	4.99 ± 0.08	5.21 ± 0.09	4.3
Cr	51.47 ± 9.87	59.21 ± 1.08	14.0
Cu	272.26 ± 10.90	315.11 ± 9.48	14.6
Ni	79.44 ± 4.72	80.06 ± 0.93	0.8
Pb	42.47 ± 3.78	48.13 ± 1.51	12.5
Zn	811.97 ± 14.50	767.50 ± 12.31	5.6

Results are expressed as the mean ($\bar{x}$) ± standard deviation (SD) in mg·kg^−1^ of dry matter (DM).

**Table 8:** The percentage differences between the content of heavy metals in the two methods of sequential extraction in dewatered sewage sludge.

HM	Sum of the 4 Chemical Fractions		Percent Difference between CSE and USE
	Conventional Extraction (CSE)	Ultrasound-Assisted Extraction (USE)	
	mg·kg^−1^		%
Cd	4.60 ± 0.31	4.88 ± 0.14	5.9
Cr	73.04 ± 5.27	76.05 ± 1.82	4.0
Cu	243.15 ± 10.99	244.99 ± 3.99	0.8
Ni	157.65 ± 2.03	153.27 ± 3.04	2.8
Pb	61.95 ± 3.46	67.60 ± 0.10	8.7
Zn	1162.20 ± 12.37	1112.35 ± 16.00	4.4

Results are expressed as the mean ($\bar{x}$) ± standard deviation (SD) in mg·kg^−1^ of dry matter (DM).

## Data Availability

Not applicable.

## Sample Availability

Samples of the compounds are not available from the authors.
